# Highly Continuous Genome Assembly of Eurasian Perch (*Perca fluviatilis*) Using Linked-Read Sequencing

**DOI:** 10.1534/g3.118.200768

**Published:** 2018-10-24

**Authors:** Mikhail Yu. Ozerov, Freed Ahmad, Riho Gross, Lilian Pukk, Siim Kahar, Veljo Kisand, Anti Vasemägi

**Affiliations:** * Department of Biology, University of Turku, 20014, Finland; § Institute of Technology, Faculty of Science and Technology, University of Tartu, Tartu, 50411, Estonia; † Chair of Aquaculture, Institute of Veterinary Medicine and Animal Sciences, Estonian University of Life Sciences, Tartu, 51014, Estonia; ‡ Department of Fisheries and Wildlife, Michigan State University, Michigan, 48824; ** Department of Aquatic Resources, Institute of Freshwater Research, Swedish University of Agricultural Sciences, Drottningholm, 17893, Sweden

**Keywords:** *Perca fluviatilis*, whole genome sequencing, *de novo* assembly, 10X Genomics Chromium linked-read, fish

## Abstract

The Eurasian perch (*Perca fluviatilis*) is the most common fish of the Percidae family and is widely distributed across Eurasia. Perch is a popular target for professional and recreational fisheries, and a promising freshwater aquaculture species in Europe. However, despite its high ecological, economical and societal importance, the available genomic resources for *P. fluviatilis* are rather limited. In this work, we report *de novo* assembly and annotation of the whole genome sequence of perch. The linked-read based technology with 10X Genomics Chromium chemistry and Supernova assembler produced a draft perch genome ∼1.0 Gbp assembly (scaffold *N*_50_ = 6.3 Mb; the longest individual scaffold of 29.3 Mb; BUSCO completeness of 88.0%), which included 281.6 Mb of putative repeated sequences. The perch genome assembly presented here, generated from small amount of starting material (0.75 ng) and a single linked-read library, is highly continuous and considerably more complete than the currently available draft of *P. fluviatilis* genome. A total of 23,397 protein-coding genes were predicted, 23,171 (99%) of which were annotated functionally from either sequence homology or protein signature searches. Linked-read technology enables fast, accurate and cost-effective *de novo* assembly of large non-model eukaryote genomes. The highly continuous assembly of the Eurasian perch genome presented in this study will be an invaluable resource for a range of genetic, ecological, physiological, ecotoxicological, functional and comparative genomic studies in perch and other fish species of the Percidae family.

The Eurasian perch, *Perca fluviatilis* (NCBI Taxon ID: 8168, Fishbase ID: 358) is a common medium-size predatory fish of the Percidae family that is widely distributed across northern Eurasia. It can live in an extremely broad range of habitats, from estuarine lagoons and lakes of all types to rivers and medium-sized streams. Perch is an important commercially exploited fresh- and brackish water fish species, and also a very popular target among recreational fisherman. As the current demand of perch exceeds fisheries production, it has been introduced as a new aquaculture species in many European countries (Policar *et al.* 2015).

Although a number of phylogeographic, population genetic and genomic studies have been conducted in perch using mtDNA, microsatellites and RAD-seq (*e.g.*, Nesbø *et al.* 1999; Gerlach *et al.* 2001; Bergek *et al.* 2010; Bergek and Björklund 2009; Olsson *et al.* 2011; Pukk *et al.* 2013; Pukk *et al.* 2015; Pukk *et al.* 2016), current genomic resources of *P. fluviatilis* are rather limited. Recently, several RNAseq studies have been performed on perch to generate *de novo* transcriptome assemblies (Pasquier *et al.* 2016; Chen *et al.* 2017) and a *de novo* genome draft assembly of perch was published among those of 66 teleost fishes (Malmstrøm *et al.* 2017). However, this genome draft assembly is very fragmented and incomplete (scaffold *N*_50_ = 5,973 bp) which severely limits its usefulness for subsequent genomic work.

Modern technologies of next-generation sequencing enable the generation of billions of short reads with high accuracy for a relatively low price (Levy and Myers 2016). However, *de novo* assembly of a genome using short reads is challenging; obtaining long continuous scaffolds is difficult, as short reads perform poorly for resolving repetitive structures or GC-biased regions (Sohn and Nam 2018). These challenges arising from the complexity of genome structure can be overcome by using single-molecule long reads, but the error rate and the sequencing cost for these long-read technologies remain high (Sohn and Nam 2018). A library preparation technology developed by 10X Genomics incorporates unique molecular barcodes into individual high molecular weight DNA molecules, after which libraries undergo standard Illumina short-read sequencing (Zheng *et al.* 2016). A phased assembly strategy algorithm implemented in Supernova software then uses these barcodes to tag short-reads that come from the same long DNA fragment (linked-reads), enabling the construction of highly continuous scaffolds (Weisenfeld *et al.* 2017). 10X Genomics linked-read sequencing has been successfully applied to generate *de novo* genome assemblies of several organisms including plants (Hulse-Kemp *et al.* 2018; Liu *et al.* 2018), amphibians (Hammond *et al.* 2017), mollusks (Li *et al.* 2018) and mammals (Jones *et al.* 2017; Mohr *et al.* 2017).

Here, we report a high-quality, highly continuous, and nearly complete assembly of the Eurasian perch genome generated using 10X Genomics linked-read sequencing, which will serve as a backbone for future genetic, genomic, ecological and evolutionary studies of perch and other fish species of the Percidae family.

## Materials and Methods

### Samples, library preparation and sequencing

A single female perch from the small humic lake Loosalu, Estonia (58.932°N 25.080°E; lake surface area 35.2 ha) was caught by gill-net on 19.06.2017 and killed by an overdose of tricaine methanesulfonate (MS-222) before sampling. A blood (350 µl) sample was collected, mixed with 15 µl of K_2_EDTA, and kept on ice during transportation to the laboratory. Buffered blood was kept at +4° and DNA isolation was carried out on the third day after sample collection. Transcriptome characterization was performed using a different female perch, caught one year earlier (16.09.2016) from the same lake and killed as described above. A whole left eyeball was dissected from this perch and immediately stored in liquid nitrogen. The permits for sample collection were issued by the Estonian Ministry of the Environment (no. 54/2016; 37/2017).

High molecular weight genomic DNA (gDNA) was isolated from blood using the MagAttract HMW DNA Kit (Qiagen, Halden, Germany) according to manufacturer instructions with a few modifications. As fish red blood cells contain a nucleus, we used only 1 µl of buffered blood (instead of recommended 200 µl). In addition, to avoid fragmentation of high molecular weight DNA, we applied very gentle vortexing and mixing during the DNA isolation procedure. Total DNA was eluted in 80 µl of double distilled water. The quantity of gDNA was measured by Qubit Fluorometric Quantitation (Life Technologies) and the average length of the gDNA fragments was determined using the Agilent 2200 TapeStation system using Genomic DNA ScreenTape (cat. 5067-5365) and Genomic DNA Reagents (cat. 5067-5366). The average fragment size of gDNA was > 60 Kb. Genomic DNA was adjusted to a concentration of 0.6 ng/µl and 0.75 ng of template gDNA was loaded on a Chromium Genome Chip. Whole genome sequencing libraries were prepared using Chromium Genome Library & Gel Bead Kit v.2 (10X Genomics, cat. 120258), Chromium Genome Chip Kit v.2 (10X Genomics, cat. 120257), Chromium i7 Multiplex Kit (10X Genomics, cat. 120262) and Chromium controller according to manufacturer’s instructions. Briefly, gDNA was combined with Master Mix, a library of Genome Gel Beads, and partitioning oil to create Gel Bead-in-Emulsions (GEMs) on a Chromium Genome Chip. The GEMs were isothermally amplified with primers containing an Illumina Read 1 sequencing primer, a unique 16-bp 10x bar-code and a 6-bp random primer sequence, and bar-coded DNA fragments were recovered for Illumina library construction. The amount and fragment size of post-GEM DNA was quantified prior to library construction using a Bioanalyzer 2100 with an Agilent High sensitivity DNA kit (Agilent, cat. 5067-4626). Quantitative polymerase chain reaction (qPCR) using KAPA Library Quantification Kit for Illumina platforms (Kapa Biosystems, cat. KK4873) was performed to assess library yield. The library size range and distribution was determined using the Fragment Analyzer Automated CE System (AATI) with a High Sensitivity NGS Fragment Analysis Kit (Advanced Analytical, cat. DNF-474-1000). The library was sequenced on two lanes of an Illumina HiSeq 2500 sequencer in rapid run mode, using paired-end sequencing to generate 580.55 M linked-reads with a mean read length of 139.5 bp after trimming. The weighted mean molecule size was estimated as 63.18 Kb and mean read coverage was ∼68x. The WGS library preparation and sequencing was performed in the Finnish Functional Genomics Centre (Turku, Finland).

For transcriptome characterization, the frozen eyeball was mechanically crushed in liquid nitrogen using a mortar and pestle to produce a fine powder. Total RNA was extracted from the whole homogenized eyeball (30 mg of tissue), using a NucleoSpin RNA extraction kit (MACHEREY-NAGEL, Duren, Germany). The quality of the total RNA sample was evaluated using Bioanalyzer 2100 (Agilent) electrophoresis and sample concentration was measured with a Nanodrop ND‐2000 (Thermo Scientific). The library was prepared from 300 ng of total RNA according to the Illumina TruSeq Stranded mRNA Sample Preparation Guide (part no. 15031047) to generate a 300-bp insert size library. The library was sequenced using an Illumina HiSeq 3000 (2 × 75 bp configuration, half a lane) in the Finnish Functional Genomics Centre (Turku, Finland).

### Evaluation of the genome metrics based on raw reads

K-mer counting of quality and barcode trimmed Illumina reads was performed using Jellyfish v.2.2.6 (Marçais and Kingsford 2011), producing k-mer frequency distributions of 17-, 21- and 25-mers (jellyfish histo -h 3000000). These histograms were processed using GenomeScope (Vurture *et al.* 2017; high frequency k-mer cutoff = 10,000), and findGSE (Sun *et al.* 2018) to estimate genome size, heterozygosity and repeat content.

### de novo genome assembly

The linked-read data were assembled using Supernova v.2.0.1 assembler (Weisenfeld *et al.* 2017) with default settings. The assembler software was run for 25 days on a 28 core and 240 Gb RAM CSC – IT Center for Science cPouta virtual private server, based on Intel Xeon CPU E5-2680 v.4 2.4 GHz processors. The initial draft genome assembly was presented in pseudohaplotype format, and contained 1,024.4 Mb of scaffold sequence (37,560 scaffolds ≥ 1 Kb), of which 111.5 Mb represented unknown bases. GenomeTools sequniq v.1.5.10 (Gremme *et al.* 2013) was applied to remove duplicated scaffolds (1,374 scaffolds) and only scaffolds with more than 10% of unique sequence were retained (36,169 scaffolds). The redundancy of the genome assembly was further reduced in two steps. First, all scaffolds < 2 Mb were clustered using CD-HIT v.4.7 package (Fu *et al.* 2012; Li and Godzik 2006). When two or more scaffolds showed ≥ 99% similarity, all but the longest scaffold were removed to generate a non-redundant set of scaffolds < 2 Mb. This resulted in removal of 4,396 potentially redundant scaffolds from the assembly. Second, to further reduce potential redundancy, the assembly including non-redundant set of < 2 Mb scaffolds was self-aligned using LAST v.926 (Kiełbasa *et al.* 2011; identity ≥ 99%, coverage of query sequence ≥ 95%), resulting in exclusion of 668 additional scaffolds. In total, 66,188,489 bp were removed from the initial assembly due to potential duplication or redundancy. It should be noted that the size of the majority of potentially redundant scaffolds did not exceed 10 Kb, varying from 1 Kb to 621.4 Kb (Figure S1).

The final perch genome assembly included 31,105 scaffolds. The assembly was screened for vectors and contaminants using a Kraken v.1.0 (Wood and Salzberg 2014) customized database, which included standard Kraken viral, bacterial, archaeal, plasmid and human databases, additional genomes of *Trypanosoma brucei* (GCF_000210295.1, Jackson *et al.* 2010) and seven fish species (*Cyprinus carpio* GCF_000951615.1, Li J.-T., Chinese Academy of Fishery Science; *Danio rerio* GCF_000002035.6, Howe *et al.* 2013; *Esox lucius* GCF_000721915.3, Rondeau *et al.* 2014; *Lates calcarifer* GCF_001640805.1, Vij *et al.* 2016; *Nothobranchius furzeri* GCF_001465895.1, Senf *et al.*, Leibniz Institute for Age Research – Fritz Lipmann; *Oncorhynchus mykiss* GCF_002163495.1, Lien *et al.*, Norwegian University of Life Sciences; and *Takifugu rubripes* GCF_000180615.1, Kai *et al.* 2011). In total, 71 and 472 scaffolds were detected as potentially contaminated by unicellular organisms or human DNA, respectively. NCBI’s blastn v.2.6.0 (Boratyn *et al.* 2013) was further applied to align those scaffolds to viral, bacterial, trypanosoma or to human refseq gene sequences. As the majority of the significant hits did not cover more than 1% of query sequence, all of the scaffolds were considered as non-contaminated and were retained for further analyses.

QUAST v.4.5 (Gurevich *et al.* 2013) was utilized to generate metrics for genome assembly and to compare it with the previously published *P. fluviatilis* assembly by Malmstrøm *et al.* (2017). Genome assembly completeness was estimated with BUSCO v.3.0 (Simão *et al.* 2015) using a ray-finned fishes (*Actinopterygii* obd9) database consisting of 4,584 orthologs from 20 fish species.

### Transcriptome assembly

To assist in the subsequent genome annotation, we performed RNA sequencing and *de novo* transcriptome assembly, which was used to complement a perch transcriptome assembly published earlier (Pasquier *et al.* 2016). A total of 526 M reads were generated. Short (< 50 bp) and low quality reads (average quality ≤ 25) were trimmed using Trimmomatic v.0.35 (Bolger *et al.* 2014; SLIDINGWINDOW:5:25 MINLEN:50). rCorrector (Song and Florea 2015) was applied to correct random sequencing errors and remove erroneous k-mers from Illumina paired-end reads. Further, to reduce bias in downstream analyses due to over ribo-depletion (Lahens *et al.* 2014) the corrected trimmed reads were mapped to an rRNA database (SILVA Release 128; Pruesse *et al.* 2007). Finally, 419 M filtered reads were assembled *de novo* using Trinity v.2.3.2 (Haas *et al.* 2013) with default parameters. As our *de novo* transcriptome assembly was based only on eye tissue and its estimated BUSCO completeness was 79.1%, we combined it with the multi-tissue transcriptome assembly of perch published earlier (Pasquier *et al.* 2016) following the protocol described in Cerveau and Jackson (2016). The redundancy of the combined transcriptome assembly was further reduced using CD-HIT v.4.7 (Fu *et al.* 2012; Li and Godzik 2006). When two or more transcripts showed 90% or higher similarity all but the longest transcript were removed to generate non-redundant set of transcripts.

### Repeat-content analysis

To identify repeats in the genome assembly, a *de novo* repeat library was first built based on the large scaffolds (≥ 10 Kb) using RepeatModeler v.1.0.11 (Smit and Hubley 2008-2015) with default parameters. The screening for repeats and low complexity sequences in the assembly was performed in RepeatMasker v.4.0.7 (Smit *et al.* 2013-2015) using *de novo* repeat library in combination with Dfam consensus 20171107 (Hubley *et al.* 2016) and RepBase 20170127 (Bao *et al.* 2015) repeat libraries.

### Gene prediction and annotation

Gene models were constructed with MAKER v.2.31.8 (Holt and Yandell 2011), which incorporates *ab initio* gene prediction, homology-based prediction and RNA-seq assisted prediction. Prior to *ab initio* gene prediction, repeat regions of the perch genome were masked based on repeat annotation results. A total of three MAKER runs were performed. First, protein sequences from 11 other fish species from the Ensembl 91 database and the combined set of perch transcripts were aligned to the genome in an initial MAKER run as evidence to retrain Augustus v.3.2.2 (Stanke *et al.* 2006) and SNAP v.2006-07-28 (Korf 2004) *ab initio* gene prediction tools. The second and third runs of MAKER included gene models trained from the first (and then second) runs with *ab initio* gene prediction tools. Augustus was retrained within the BUSCO v.3.0 pipeline using genomic regions containing mRNA annotations from initial (and then second) MAKER run (including additional 1,000 bp on each side). BUSCO runs were performed using the –long option to optimize the HMM settings of the raw zebrafish HMM (–sp zebrafish; first run) or trained perch HMM (second run) and to generate the final trained perch HMM. Retraining of SNAP was performed using gene models from the initial (and then second) MAKER run with an annotation edit distance (AED) ≤ 0.25 and a length of amino acids ≥ 50. AED ranges from 0 to 1 and quantifies the congruency between a gene annotation and its supporting evidence (EST, protein and mRNA-seq alignments). Lower AED values imply higher congruency between the intron-exon coordinates of an annotation and its aligned evidence, whereas AED = 1 indicates no evidence for support of predicted genes. Only sequences with AED < 0.5 and CDS ≥ 90 bp were retained in the final set of predicted genes.

NCBI’s blastp v.2.6.0 (Boratyn *et al.* 2013; -evalue 1e-10, -soft_masking true, -lcase_masking, and a hit fraction filter to include only hits of > 70% target length, -qcov_hsp_perc 70) was used to functionally annotate the genes against vertebrate sequences in the NCBI non-redundant database. Further, non-annotated sequences were searched against all sequences in the NCBI non-redundant database. In addition, protein motifs, domains and signatures present in the predicted protein sequences were annotated using Interproscan v.5.24 (Jones *et al.* 2014) by searching against publicly available databases, including Pfam (Finn *et al.* 2014), PRINTS (Attwood *et al.* 2012), PROSITE (Sigrist *et al.* 2013), SMART (Letunic *et al.* 2012), SUPERFAMILY (de Lima Morais *et al.* 2011), and TIGRFAMs (Haft *et al.* 2013).

### Data availability

Short Illumina linked-reads are available in the NCBI Sequence Read Archive (SRA; SRR7091761), and the Whole Genome Assembly has been deposited at DDBJ/EMBL/GenBank under the accession QFAT00000000, both under BioProject PRJNA450919. Transcriptome reads are available in the NCBI SRA (SRR7091762), and the eye Transcriptome Shotgun Assembly has been deposited at DDBJ/EMBL/GenBank under accession number GGNF00000000, as part of BioProject PRJNA450919. The combined transcriptome assembly from multiple tissues and Figure S1 have been uploaded as supplementary file to Figshare: https://doi.org/10.25387/g3.7156304.

## Results and Discussion

### Genome characteristics

Genome size estimates from GenomeScope ranged from 851.7 to 928.2 Mb, whereas estimates based on findGSE (fitted and original counts with corrected k-mer coverage) were higher and ranged from 1,050.9 Mb to 1,172.8 Mb (Table 1). Genome size estimates from both methods were in reasonable agreement with those determined earlier using cell flow cytometry (880.2 – 1,193.2 Mb; Vialli 1957; Vinogradov 1998). The analysis using GenomeScope indicated low heterozygosity (0.24–0.28%) in comparison with other species (*e.g.*, Kajitani *et al.* 2014; Vurture *et al.* 2017). Low heterozygosity of the sequenced individual is also consistent with population genetic data from 16 perch populations screened using microsatellite markers, where lake Loosalu perch population showed the lowest level of genetic diversity (A. Vasemägi, unpublished results). Similar to other freshwater Perciformes (Yuan *et al.* 2018), the estimated proportion of repeats in perch was relatively high, ranging from 33.1% (k = 25, GenomeScope) to 55.0% (k = 17, findGSE).

**Table 1:** Genome size, heterozygosity and repeat content as estimated by GenomeScope and findGSE software

Genome characteristics	k-mer size		
	k = 17	k = 21	k = 25
*GenomeScope*			
Genome haploid length (Mb)	851.7	894.8	928.2
Genome repeat length (Mb)	426.9	306.9	307.4
Genome unique length (Mb)	424.8	587.9	620.8
Heterozygosity, %	0.28	0.26	0.24
Estimated repetitive ratio,%	50.1	34.3	33.1
Read error rate, %	0.14	0.18	0.19
			
*findGSE*			
Genome haploid length (Mb)	1,050.9	1,163.8	1,172.8
Genome repeat length (Mb)	578.1	529.8	503.2
Estimated repetitive ratio, %	55.0	45.5	42.9

### Genome assembly

**Figure 1:**
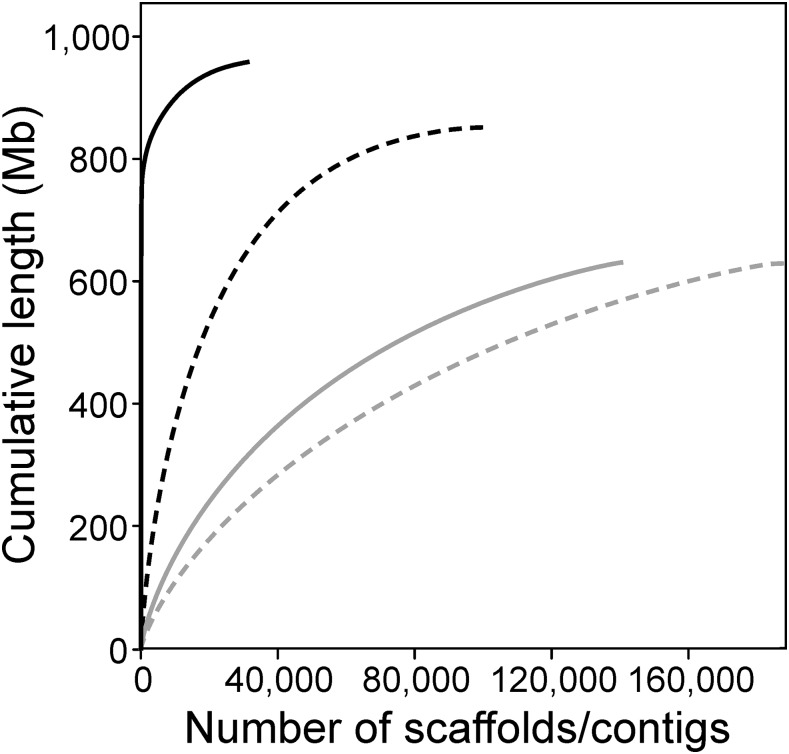
Cumulative length of the assembly represented by scaffolds (solid line) and contigs (dashed line). *De novo* perch genome assembly obtained using linked-reads (this study, black lines) and recently published genome assembly using Illumina short reads (Malmstrøm *et al.* 2017, gray lines).

The total length of the assembly was 958.2 Mb, which included 106.6 Mb of unknown bases. The high number of unknown bases is typical for the Supernova assembler (*e.g.*, Mohr *et al.* 2017; Hulse-Kemp *et al.* 2018), as it estimates gap sizes rather than introducing an arbitrary value of Ns during scaffolding (Weisenfeld *et al.* 2017). In the presented perch genome assembly, repeat regions were estimated to account for 32.72% (281.6 Mb). The contig *N*_50_ and scaffold *N*_50_ sizes were 18.2 Kb and 6.3 Mb, respectively (Table 2). More than 80% of the assembly was covered by the 516 longest scaffolds (≥ 50 Kb; 1.7% of all scaffolds). Compared to the draft perch genome assembly published by Malmstrøm *et al.* (2017) the contig and scaffold continuity (*N*_50_) metrics were improved by four and 1048 times, respectively (Figure 1, Table 2). The overall assembly size increased from 630.6 Mb to 958.2 Mb in comparison with the genome assembly by Malmstrøm *et al.* (2017) and was close to the estimates based on k-mer frequency distributions or cell flow cytometry.

**Table 2:** Eurasian perch genome assembly statistics

	10X Genome assembly*	Genome assembly by Malmstrøm *et al.* (2017)*
*Contig statistics*
Number of contigs	100,796	181,537
Total contig size (bp)	851,640,084	626,588,998
Contig *N*_50_ size (bp)	18,196	4,140
Largest contig (bp)	241,857	46,493
*Scaffold statistics*		
Number of scaffolds	31,105	139,898
Total scaffold size (bp)	958,225,764	630,583,430
Scaffold *N*_50_ size (bp)	6,260,519	5,973
Largest scaffold (bp)	29,260,448	73,288
GC/N (%)	40.9/11.1	40.6/0.6
*BUSCO genome completeness*		
Complete	4,033 (88.0%)	2,144 (46.8%)
Complete and single copy	3,933 (85.8%)	2,105 (45.9%)
Complete and duplicated	100 (2.2%)	39 (0.9%)
Fragmented	323 (7.0%)	1246 (27.2%)
Missing	228 (5.0%)	1194 (26.0%)
*Annotation*		
Number of protein-coding genes	23,397	
Number of functionally-annotated proteins	23,171	
Mean protein length (interquartile range, aa)	506 (224-614)	
Longest protein (aa)	8,907 (nesprin-1)	
Average number (length, interquartile range of length) of exon per gene	9 (228, 89-189 bp)	
Average number (length, interquartile range of length) of intron per gene	8 (1,224, 150-1,340 bp)	

* Minimum scaffold length is 1 Kb.

Our perch genome assembly covered 88.0% complete and 7.0% partial ray-finned fishes BUSCOs, showing a substantial increase in completeness compared to the genome assembly by Malmstrøm *et al.* (2017) (46.8% complete and 27.2% partial BUSCOs).

### Transcriptome assembly

The final concatenated perch transcriptome assembly based on multiple tissues consisted of 36,431 transcripts covering 96.2% complete and 1.3% partial ray-finned fish benchmarking universal single-copy orthologs (BUSCOs). The total transcriptome size was 108.7 Mb and the *N*_50_ transcript size was 3.9 Kb (Table 3).

**Table 3:** Eurasian perch transcriptome assembly statistics

	Combined transcriptome assembly (multiple tissues)
*Transcript statistics*	
Number of transcripts	36,431
Total transcript size (bp)	108,727,847
Transcript *N*_50_ size (bp)	3,962
Largest transcript (bp)	78,856
*BUSCO transcriptome completeness*	
Complete	4,411 (96.2%)
Complete and single copy	3,644 (79.5%)
Complete and duplicated	767 (16.7%)
Fragmented	58 (1.3%)
Missing	115 (2.5%)

### Genome annotation

The final annotation of the *P. fluviatilis* genome from the MAKER annotation pipeline included 23,397 protein-coding genes (Table 2). NCBI’s blastp resulted in putative function annotation of 21,997 proteins (94.0%) based on homology. Further, Interproscan detected motifs, domains and signatures for 22,426 proteins (95.8%). As a result, 23,171 genes were annotated by at least one of the two methods (blastp 94.0%, InterProScan 95.8%), accounting for about 99.0% of the genes of *P. fluviatilis* (Table 2).

### Conclusions

10X Genomics linked-read technology combined with low error rate short-read sequencing enabled accurate and more continuous *de novo* assembly of the genome of Eurasian perch. A large set of annotated genes with known homology revealed in our study (21,997) will ease further gene ontology and functional genomic analyses. In addition, improved scaffold length will facilitate detection of SNPs and structural variants, such as large insertions/deletions and copy number variations, potentially responsible for adaptation of *P. fluviatilis* to various environments. While a relatively large proportion of repeat regions in the perch genome still remain unresolved, generated short reads will be useful for future analyses of repetitive DNA elements. Taken together, the highly continuous assembly of the Eurasian perch genome presented in this study will serve as an invaluable resource for a range of genetic, ecological, physiological, ecotoxicological, functional and comparative genomic studies in perch and other fish species of the Percidae family.
